# MXenes: Manufacturing, Properties, and Tribological Insights

**DOI:** 10.3390/ma18173927

**Published:** 2025-08-22

**Authors:** Subin Antony Jose, Alessandro M. Ralls, Ashish K. Kasar, Alexander Antonitsch, Daniel Cerrillo Neri, Jaybon Image, Kevin Meyer, Grace Zhang, Pradeep L. Menezes

**Affiliations:** Department of Mechanical Engineering, University of Nevada-Reno, Reno, NV 89557, USA; subinj@unr.edu (S.A.J.); alessandroralls@gmail.com (A.M.R.); ashishkasar91@gmail.com (A.K.K.); alexander_antonitsch@unr.edu (A.A.); jaybon.image@gmail.com (J.I.); kevinmeyer@unr.edu (K.M.); mengjunz@unr.edu (G.Z.)

**Keywords:** MXene, MAX phase, surface functionalization, chemical etching, tribological properties

## Abstract

MXenes, a novel class of two-dimensional (2D) transition metal carbides and nitrides, have garnered significant attention due to their exceptional thermal conductivity, electrical properties, and mechanical strength. This review offers a comprehensive overview of MXenes, focusing on their synthesis methods, material properties, tribological performance, and potential challenges and opportunities. Typically synthesized through the selective etching of layered precursors, MXenes offer highly tunable structures, allowing for precise tailoring for specific functionalities. Their outstanding properties, such as high electrical conductivity, chemical versatility, mechanical durability, and intrinsic lubricity, make them promising candidates for various applications, including energy storage, electromagnetic shielding, water purification, biosensing, biomedicine, and advanced tribological systems. While many of these applications are briefly acknowledged, this review primarily emphasizes MXenes’ potential in tribological applications, where recent studies have highlighted their promise as solid lubricants and tribological additives due to their low shear strength, layered structure, and ability to form protective tribofilms under sliding contact. However, challenges such as oxidation resistance, long-term stability, and performance under extreme environments continue to impede their full potential. With less than a decade of focused research, the field is still evolving, but MXenes hold tremendous promise for revolutionizing modern material science, especially in next-generation lubrication and wear-resistant systems. This review explores both the opportunities and challenges associated with MXenes, emphasizing their emerging role in tribology alongside their broader engineering applications.

## 1. Introduction

**Figure 1:**
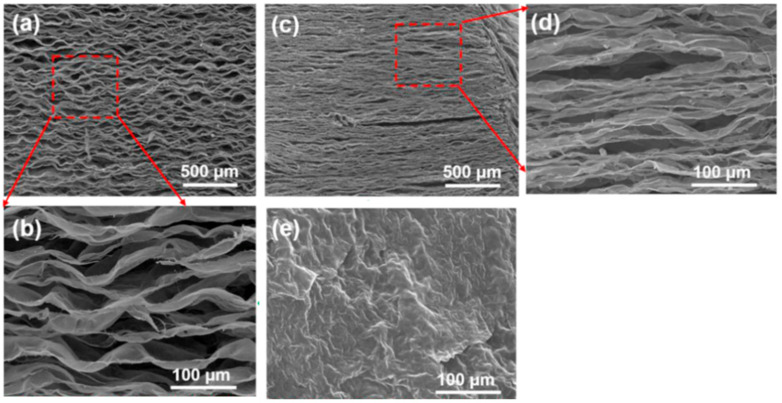
Electron microscopy images of the 2D lamellar structure of MXenes from the (**a**,**b**) X-Z, (**c**,**d**) X-Y, and (**e**) Y-Z planes, adapted with permission from [3].

In today’s day and age, advancements in technology and science have led to the creation of new and innovative inventions that continue to shape the world positively. A large portion of these inventions is usually material-based, with a focus on improving the performance of key components in diverse operational environments. Amongst the variety of these inventions, a relatively new class of two-dimensional (2D) material known as MXenes has attracted a large amount of research and industrial attention in recent years. Being composed of early transition metals (i.e., “M”, which is composed of either Cr, Zr, Tu, V, Nb, Mn, Mo, or Ta) and carbon and/or nitrogen (i.e., “X”), MXenes take on a lamellar-type structure (as shown in Figure 1), which enables unique properties that allow for their usage in a wide variety of applications [1,2].

**Figure 2:**
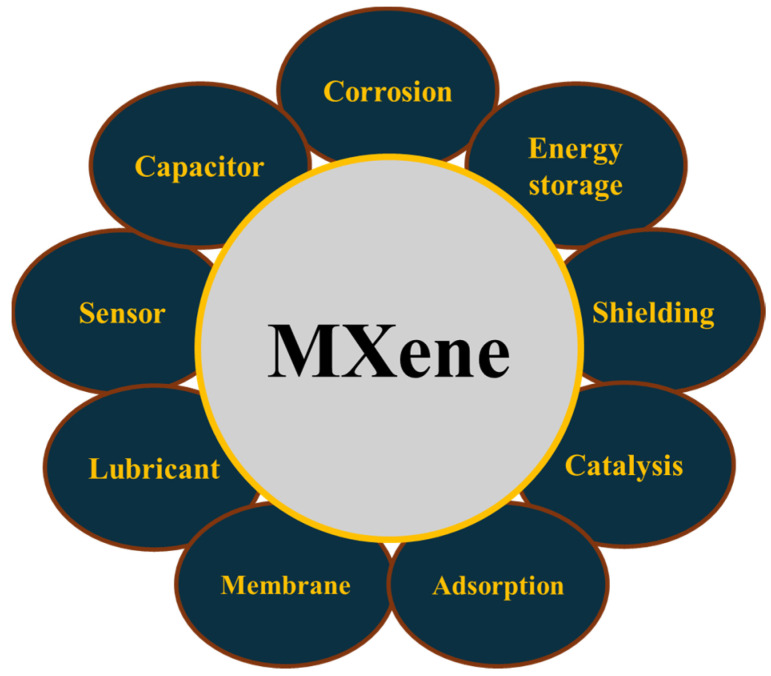
A visualization of the various applications in which MXenes are used.

Generally speaking, 2D materials themselves have an upper hand in mechanical and electrical applications as they exhibit characteristics such as enhanced flexibility, large band gaps, the ability to become extremely thin, and an exceptional surface/volume ratio [4]. These traits are desirable to industries that require highly effective and sustainable materials. Among the various applications in which MXenes are used, they are employed in energy storage devices, sensors, detectors, nanotechnology, and as composites in mechanical, tribological, and corrosion-based materials, as illustrated in Figure 2 [4,5,6,7,8,9,10].

Ajmal et al. [11] provide a comprehensive review of MXenes, emphasizing their role across diverse application areas. The article offers an in-depth discussion on the development of MXene-based composites incorporating carbon materials, polymers, and metal oxides. It also highlights their exceptional performance in supercapacitors, batteries, environmental remediation, and electromagnetic interference (EMI) shielding, with a strong emphasis on application-driven insights.

To assess the rising research interest in MXenes, a comprehensive analysis of publicly available sources, such as journal articles, books, book chapters, and patents, was conducted using the Web of Science database [12]. The search criteria that are detailed in Table 1 were applied, and the resulting publication counts were used as a measure of the research activity of this article.

**Figure 3:**
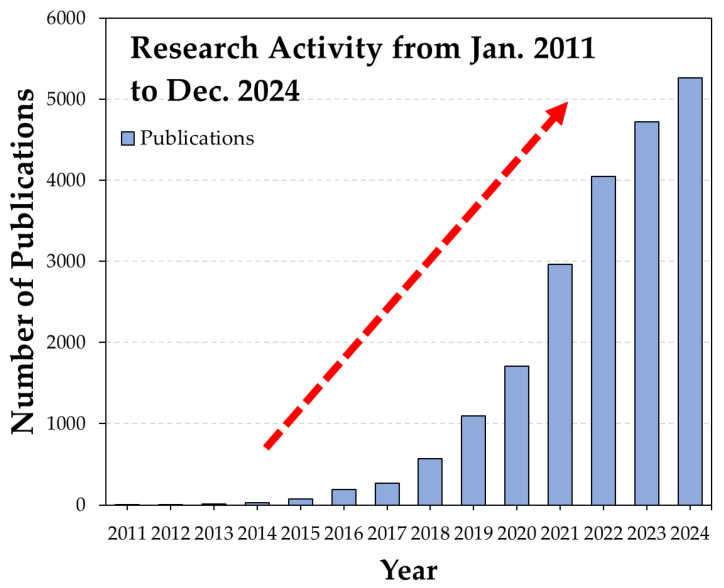
The increase in the number of publications for the MXene-related literature from January 2011 to December 2024, provided by the Web of Science [12].

The results of this systematic analysis are presented in Figure 3 and reveal a steady and significant increase in MXene-related publications since their discovery in 2011 [13]. With research activity showing no signs of slowing, this review aims to present a comprehensive examination of recent advancements in the field of MXenes from multiple perspectives. It begins with an introduction to MXenes, followed by an overview of the synthesis techniques employed in their production. The review then explores the key properties of MXenes, particularly their mechanical, electrical, and thermal characteristics. Although various applications of MXenes are briefly introduced, this review places a focused emphasis on tribological applications. This includes an in-depth discussion on their performance as solid lubricants and tribological additives, which is an area of growing interest, and yet is relatively underrepresented in the current literature. This review concludes with a discussion on future directions, including challenges related to synthesis, stability, large-scale implementation, and environmental considerations. Overall, this work serves as a critical reference for researchers and engineers seeking to understand and advance the use of MXenes in diverse technological domains.

## 2. Overview of MXenes

### 2.1. What Are MXenes and Why Do They Matter?

MXenes are a new class of 2D materials that have shown great potential in a variety of fields in recent years. MXenes were first discovered in 2011 by researchers at Drexel University [13,14]. However, it was not until 2017 that research in MXenes truly grew. After this year, research on MXenes in a variety of applications, including but not limited to ultrahigh-temperature ceramics, bone regeneration, water treatment, and environmental remediation, began to explode [15,16,17]. After 13 years of development, there are up to 46 different types of MXenes that have been created in the laboratory, and more than 100 theoretical predictions have been made, including those with in-plane and out-of-plane ordering of metal atoms [18,19].

**Figure 4:**
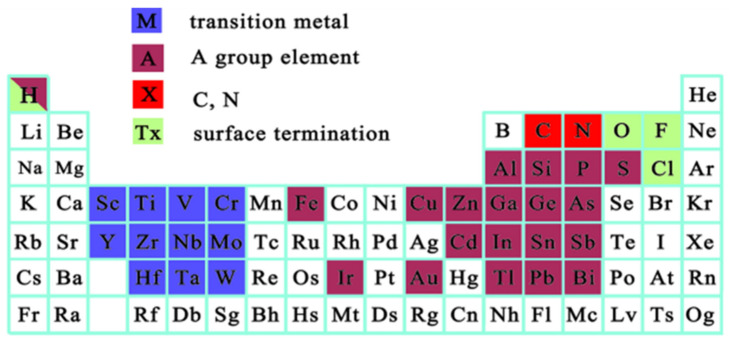
The elements that correspond to the MAX phase. Reproduced from [26], open access.

As previously mentioned, MXenes are a group of early transition metal carbides and/or carbonitrides. Traditionally, MXenes are chemically denoted as M_n+1_X_n_T_x_, with n being from 1 to 4 [6]. As shown in Figure 4, M represents an early transition metal, X represents carbon and/or nitrogen, and T_x_ is the corresponding functional (or frequently referred to as a surface termination) group, typically composed of hydroxyl (-OH), fluorine (-F), and/or oxygen (-O) [20,21]. Many known substances, such as graphene, hexagonal boron nitride (hBN), and molybdenum disulfide (MoS_2_), all have similar structures that contain high aspect ratios and ultrathin thicknesses corresponding to a few atomic layers [22]. The reason why MXenes are extremely important is that, as a new type of binary nanomaterial, they not only inherit many advantages of ordinary 2D nanomaterials (i.e., extreme thinness, large specific surface area, high surface-to-volume ratio, and high in-plane stiffness), but also have many unique characteristics that allow for their diverse applications [23]. These characteristics can be closely tied to their elemental composition, as they tend to have greater electrical conductivity, mechanical strength, hydrophilicity, and flexibility compared to traditional 2D materials [24,25].

### 2.2. MXene Manufacturing

Having a preliminary understanding of what MXenes are, it is crucial to understand the synthesis techniques that are used to fabricate MXenes. The following section will provide a holistic view of the approaches that are currently used for MXene fabrication. Furthermore, the microstructural features produced by the techniques will also be discussed. As a point of reference, the key findings from this section are tabulated in Table 2.

#### 2.2.1. Fluoride Etching

Across most of the literature, one of the most common techniques for fabricating MXenes is to perform chemical etching on a layered ternary material, which is frequently referred to as the MAX phase. With the M and X portions of the MAX phase representing early transition metals and C and/or N, the A portion represents an element from the A group that is interleaved within the M/X structure [52]. These phases are typically formed through powder sintering processes, which include combining and mixing different mixtures of M, A, and X [53]. When using a chemical etching approach, the interlayer “A” atoms from the MAX phase are effectively removed.

**Figure 5:**
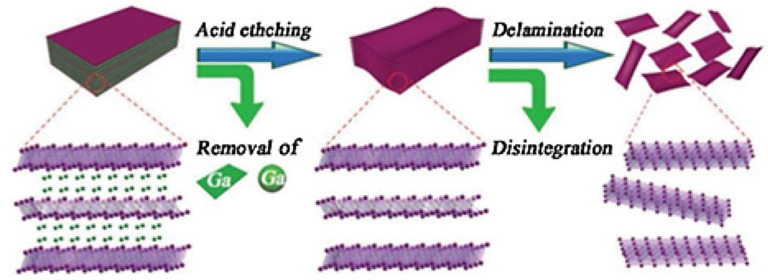
A visualization of the removal of the A element from the MAX phase from chemical-based etching. Reproduced with permission from [36].

Some selective chemical etchings include aqueous fluoride etching, which weakens the metallic bonds held in the MAX phase [17]. Others, such as Naguib et al. [54], have used techniques such as hydrofluoric acid (HF) etching, which consists of introducing/mixing HF with the MAX phase to decompose the A element. A visualization of this technique can be seen in Figure 5. When HF is introduced to the system, the A element forms H_2_, AF_3_, and M_n+1_X_n_ [28]. Eventually, the A element delaminates from the MAX structure, which results in MXenes. Alternative etching solutions that use fluoride salts/acids have also been used to develop MXenes. Common etchants include HCl/NH_4_F, HCl/LiF, H_2_SO_4_/LiF, and HCl/FeF_3_ [29,30,31,32,33,34,35,46,55].

**Figure 6:**
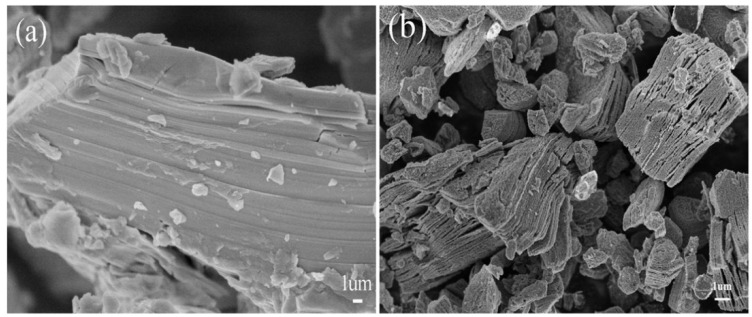
The structural features of HF-derived (**a**) Ti_3_AlC_2_/Ti_3_C_2_ and (**b**) Ti_3_AlC_2_. Reproduced with permission from [37].

From a microstructural perspective, HF-based techniques form accordion-like structures, as shown in Figure 6.

#### 2.2.2. Molten Salt Etching

**Figure 7:**
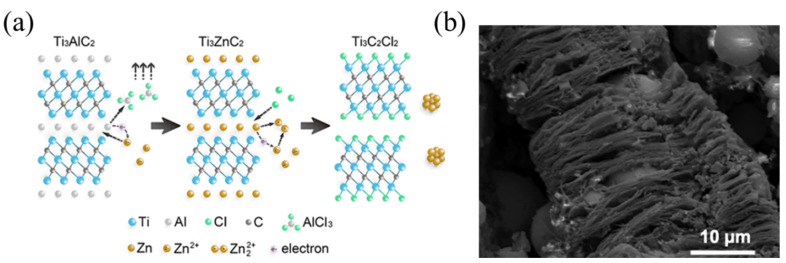
(**a**) A visualization of the etching mechanism of the MAX phase enabled by molten salt, with permission from [56]; (**b**) the microstructural accordion-like features of MXenes produced by hydrothermal etching. Reproduced with permission from [57].

Aside from the aforementioned techniques, one fluoride-based technique that has attracted a large amount of attention is molten salt etching. A visualization of this process can be seen in Figure 7a [38]. It was initially discovered by Urbankowski et al. [39], who used molten LiF/NaF/KF to form Ti_4_AlN_3_. Unlike using traditional HF techniques, this process allows for the MXene microstructure to take on an accordion-like structure (in Figure 7b), which can be advantageous for electrochemical-dependent applications (e.g., batteries, capacitors, and electrocatalysis) due to its intercalation/deintercalation abilities [40,41,42,43,44,45].

#### 2.2.3. Electrochemical Etching

**Figure 8:**
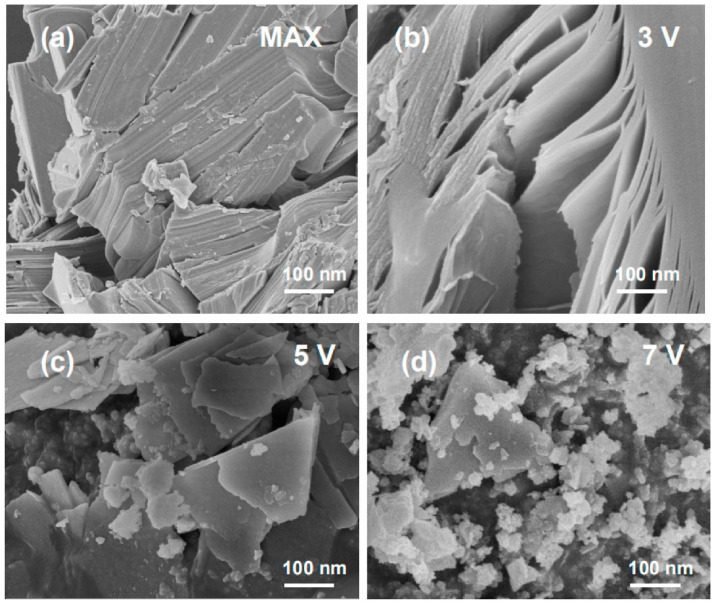
The structural features of the (**a**) MAX phase before electrochemical etching, and the MXene formed after (**b**) 3 V, (**c**) 5 V, and (**d**) 7 V etching. Reproduced with permission from [59].

Although fluoride-based etching techniques have proven to be a reliable approach for MXene synthesis, concerns about the environmental byproducts of HF solutions have resulted in the discovery of alternative etching techniques [40]. Amongst these techniques, one technique that has attracted a large amount of attention is electrochemical etching [58]. Unlike fluoride-dependent etching, electrochemical etching allows for greater control over the etching process. Parameters such as current, voltage, and time can allow for tailored structural properties [46]. Fundamentally, when the MAX phase is submersed in an electrochemical solution, the electrons from the solution (which tend to be Cl^−^ based) will transfer to the A element, thus resulting in its selective degradation [47]. A visualization of this process can be seen in Figure 8, which consists of the non-etched MAX phase (Figure 8a), as well as the influence of increasing voltage (Figure 8b–d). It can be seen that, at higher voltages (Figure 8d), excessive breakdown of the MXene structure can take place. However, by tailoring the voltage, a desirable structure can be obtained.

#### 2.2.4. Chemical Vapor Deposition Synthesis

**Figure 9:**
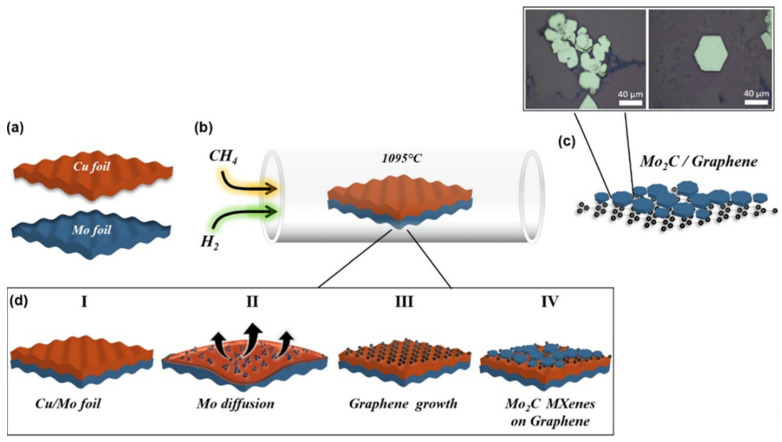
The process of MXene synthesis using CVD, including (**a**) the placement of Cu foil and Mo foil into a (**b**) quartz furnace, and (**c**) allowing for the flow of CH_4_ and H_2_ to form Mo_2_C/graphene; (**d**) a more mechanistic viewing of the synthesis process. Reproduced from [51], open access.

Up to this point, top-down approaches towards MXene fabrication have been discussed. However, aside from these approaches, the direct synthesis of MXenes can also be performed through techniques such as chemical vapor deposition (CVD) [48]. Similar to electrochemical-based techniques, the approach operates under fluoride-free conditions and can allow for the suitable control of 2D nanosheets by adjusting CVD-dependent operational parameters [49]. According to the works from Xu et al. [49] and Geng et al. [50], Mo_2_C-based MXenes can be grown, which can prove to be a viable option for MXene fabrication. For greater context on how CVD can be used for MXene fabrication, Figure 9 depicts the process from beginning to end in Figure 9a,b. Copper (Cu) and molybdenum (Mo) foils are added to a horizontal quartz furnace and exposed to CH_4_ and H_2_. After being exposed to a temperature above the melting point of Cu, Mo_2_C and graphene begin to grow (Figure 9c). This process can alternatively be seen in Figure 9d.

#### 2.2.5. Fluoride and Acid-Free Etching

There has been an increased focus on MXene synthesis strategies to develop fluoride and acid-free methods to mitigate the environmental and safety concerns associated with the conventional HF etching process. Among these, one significant contribution was made by An et al. [60] by introducing a fluoride and acid-free etching strategy for MXene synthesis. This method uses a binary alkaline solution to selectively etch the A-layer from the MAX phase, enabling the complete elimination of HF or any other strong acids. The process yields high-quality MXene with a well-obtained layered structure. Compared to traditional etching techniques, this method significantly reduces the environmental impact due to the absence of hazardous by-products [60].

Table 3 compares MXene synthesis techniques based on yield, scalability, environmental impact, and cost, as well as their advantages and limitations.

## 3. Properties of MXenes

MXenes exhibit a remarkable set of properties resulting from their unique structural and chemical characteristics. Their complex bonding, comprising both metallic and covalent bonds combined with layered atomic stacking, tunable surface terminal groups (such as -OH, -O, and -F), and diverse synthesis routes, contributes to their exceptional performance in various applications. This makes MXenes with a rare combination of ceramic-like stability and metallic conductivity, making them highly attractive for use in energy storage, sensors, catalysis, electromagnetic shielding, and tribology. Derived from ceramic MAX phases, MXenes inherit several advantageous properties, including low density, high hardness, and excellent corrosion resistance. They also exhibit metallic-like characteristics, such as high thermal and electrical conductivity and good machinability [61]. MXenes are gaining attention as promising materials for use in demanding environments. Their high electrical and thermal conductivity, along with good mechanical strength and chemical stability, make them well suited for applications like heating elements, thermal interface materials, and heat exchangers. MXenes also show potential in components such as rotating electrical contacts and bearings, where both conductivity and wear resistance are important [62]. What distinguishes MXenes from other 2D materials is their unique combination of metallic conductivity (up to 15,000 S/cm), inherent hydrophilicity (contact angle < 10°), and mechanical robustness (Young’s modulus ~0.5 TPa). These properties stem from their electronic structure, where transition metal d-orbitals enable charge transport, and their tunable surface chemistry, which facilitates covalent functionalization that is impossible in graphene [63].

### 3.1. Mechanical, Electrical, and Thermal Properties

The unique combination of mechanical, electrical, and thermal properties of MXenes sets them apart from other 2D materials and provides a broad range of advanced applications. Their high surface-area-to-volume ratio, coupled with a layered sheet-like structure, imparts excellent mechanical strength, flexibility, and tunable interlayer spacing [64,65]. These characteristics make MXenes not only robust, but also adaptable to mechanical deformation, which is especially beneficial in flexible and wearable electronics [66]. Despite their atomic-scale thickness, MXenes demonstrate mechanical properties that rival or exceed other 2D materials. The exceptional mechanical behavior stems from their unique bonding architecture; strong in-plane M-X covalent bonds provide high stiffness and strength, while weak interlayer Van der Waals interactions enable flexibility [67]. When incorporated into composite materials, MXenes provide unparalleled reinforcement effects. Just 0.5 wt% loading in polymer matrices can increase tensile strength by 200%, while ceramic composites show three times improvement in fracture toughness with MXene addition [68]. Coatings containing MXenes demonstrate an order of magnitude enhancement in wear resistance compared to conventional formulations [69]. Recent breakthroughs in MXene aerogel fabrication have yielded materials capable of withstanding 90% compressive strain while maintaining structural integrity, opening new possibilities for flexible electronics and impact-absorbing materials [70]. Due to the relatively straightforward and controllable synthesis methods, such as the selective etching of MAX phases, followed by delamination, MXenes possess the advantage of scalability. This enables the production of large quantities of MXenes materials with consistent quality, paving the way for their integration into industrial-scale technologies [25].

In terms of electrical properties, MXenes exhibit excellent conductivity due to their metallic layered structure. This, combined with their ionic mobility and hydrophilic surface terminations, makes them ideal for applications in sensors and electronic devices [70]. Given their high surface area, MXenes offer high electron mobility. Testing is being conducted to prove their effectiveness in supercapacitor applications [71]. By studying the energy of X-ray photons on the surface of MXenes, it was found that photoelectrons can be ejected from the surface but experience inelastic scattering at lower elemental composition depths [72]. This phenomenon sheds light on the complex electronic interactions occurring within MXenes and continues to spur research into their electronic structure and behavior. In other cases, MXenes have been chemically etched to have a more porous structure to provide greater electron mobility. Ti_3_C_2_T_x_ MXene has shown promise in optical detectors with memory effects, and its electrical properties are also being explored for biomedical applications [73]. MXenes also exhibit rich surface chemistry due to the presence of functional terminal groups (e.g., -OH, -O, -F), which not only affect their electronic and electrochemical properties, but also allow them to interact with other materials readily. This has led to the development of numerous MXene-based composites and hybrid structures [74].

Tian et al. studied the role of MXenes in enhancing the performance of triboelectric and piezoelectric nanogenerators. Owing to their exceptional electrical conductivity, versatile surface chemistry, and mechanical strength, MXenes facilitate efficient charge transfer and seamless structural integration. This enables their adaptability for next-generation energy-harvesting devices [75].

MXenes are also shown to play a crucial role in improving electrode design for high-performance batteries. Their unique layered structure facilitates rapid ion diffusion and high electrochemical stability, particularly in zinc-ion, lithium-ion, and sodium-ion battery systems. Additionally, their surface terminations and high conductivity help maintain capacity and structural integrity over repeated charge–discharge cycles, making them ideal candidates for advanced energy storage applications [76].

Although MXenes’ thermal properties have been less extensively studied than their electrical and mechanical characteristics, they still demonstrate remarkable thermal stability. MXenes demonstrate exceptional thermal resilience, maintaining structural integrity up to 400–800 °C in inert environments, far exceeding the stability of most 2D materials. This stability stems from their strong transition metal–carbon/nitride (M-X) covalent bonding and can be systematically tuned through surface engineering. For instance, Ti_2_N and V_2_N MXenes maintain stability over a wide temperature range [77], while Ti_3_C_2_ MXene remains stable up to 800 °C in an argon atmosphere [78]. This high-temperature resilience makes MXenes suitable for applications in energy storage, conversion, and high-temperature electronics. By integrating MXenes into hybrid systems, it is possible to fine-tune the thermal properties of the host material. However, temperature variations can affect some MXene properties, presenting challenges for certain applications.

### 3.2. Surface Chemistry and Hydrophilicity

MXenes exhibit an extraordinary degree of surface chemical control through their termination groups (-O, -OH, -F), which act as molecular dials to fine-tune material properties [79]. The specific termination profile depends critically on the synthesis route: HF etching typically produces fluorine-rich surfaces (≈60% -F), while hydrothermal methods yield hydroxyl-dominated terminations (≈80% -OH). Between these extremes, careful post-synthesis treatments can create virtually any termination ratio. Oxygen groups dramatically enhance electrical conductivity by creating electron conduction pathways, while hydroxyl groups induce extreme hydrophilicity through hydrogen bonding networks. Fluorine terminations provide exceptional environmental stability but require careful optimization as they can reduce electrochemical activity by passivating reactive sites. Advanced characterization techniques, including in situ XPS and STEM-EELS, have revealed that termination groups are not randomly distributed, but instead organize into nanoscale domains that influence macroscopic behavior [80,81,82].

MXenes also possess uniquely modifiable chemical reactivity governed by three complementary factors: (1) transition metal d-orbital electrons creating numerous accessible states near the Fermi level, (2) termination-dependent surface polarization effects, and (3) defect-mediated active sites [83]. This reactivity can be precisely controlled through surface engineering, as shown in the catalytic performance of different terminations provided in Table 4.

Recent advances have demonstrated termination-switchable MXenes that dynamically adapt their surface chemistry in response to environmental stimuli, opening new possibilities in smart catalysts and responsive materials [84].

The water affinity of MXenes stems from both their polar surface chemistry and unique layered structure, which combines surface terminations with interlayer water molecules. This dual mechanism produces some of the most hydrophilic surfaces known among 2D materials, with contact angles frequently below 5° [70]. When introduced to hydrophilic functional groups, MXenes become very chemically stable. As these composites are etched within hydrophilic solutions, MXenes can be tailored appropriately for energy storage, catalysis, and environmental remediation, like water purification. When exposed to air, many MXene powders agglomerate due to high surface energy, but hydrophilic solutions can stabilize and prevent MXenes from flaking. By using the proper solution for the MXene being studied, the uniform distribution of the MXene can be achieved. This leads on to the benefits of other characteristics of MXenes, like their electrical properties [85].

Their hydrophilic nature, due to their -OH- and -O-terminated surfaces, allows them to interact favorably with water. This makes them ideal for use in applications that require good hydrophilicity. For instance, they can be used in water purification technologies where their hydrophilic nature allows them to effectively absorb and remove contaminants from water. One common result from MXene exposure to water is the formation of stable colloidal solutions, which can be seen as MXenes that remain suspended in water and possess higher colloidal stability. MXenes also have an affinity for polar molecules, giving them desirable properties in lubrication coatings. This filtering behavior can even extend to how MXenes interact with wavelengths such as light. Hydrophilicity enables three key technological advantages: (1) spontaneous exfoliation in water without surfactants, (2) excellent interfacial adhesion in composites, and (3) compatibility with biological systems. However, for applications requiring moisture resistance, surface passivation strategies have been developed using alkyl silanes or polymer coatings that can tune wettability across a remarkable 150° range while preserving bulk properties [86].

## 4. Application of MXene in the Field of Tribology

**Figure 10:**
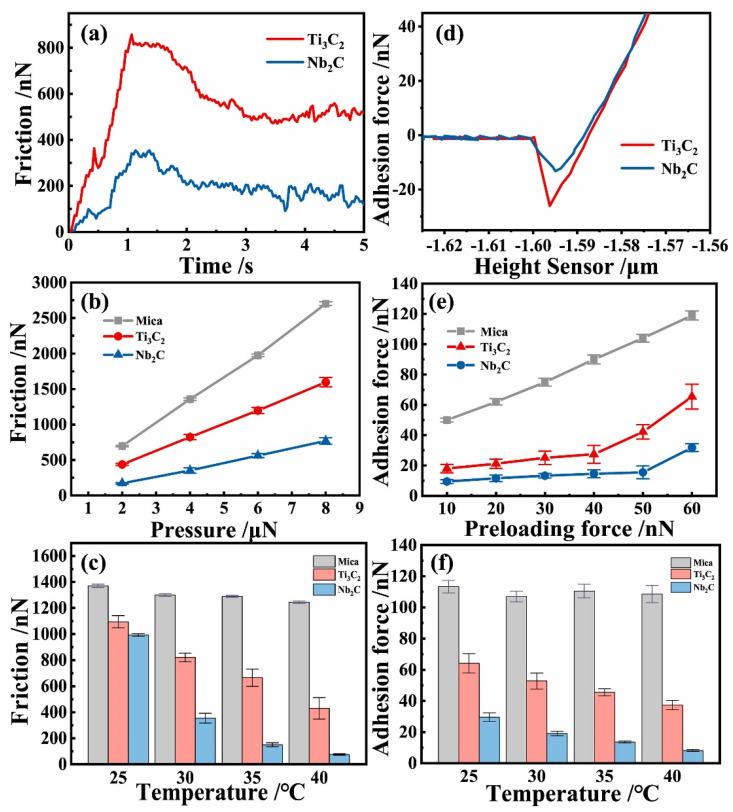
(**a**) Frictional plot of Ti_3_C_2_ and Nb_2_C at 4 μN, (**b**) Frictional plot of Ti_3_C_2_ and Nb_2_C at pressures of 2–8 μN, (**c**) Frictional plot of Mica, Ti_3_C_2_ and Nb_2_C at 4 μN at 25–40 °C, (**d**) Adhesion force curves of Mica, Ti_3_C_2_ and Nb_2_C MXene at the preloading force of 30 nN, (**e**) Adhesion force curves of Mica, Ti_3_C_2_ and Nb_2_C at the preloading force of 10–60 nN, (**f**) Adhesion force curve of Mica, Ti_3_C_2_ and Nb_2_C at the preloading force of 60 nN at 25–40 °C [87].

MXene is a new class of materials and has shown promising tribological applications. The 2D layered structure suggests lower COF, whereas the inherent hardness of carbides and nitrides provides higher wear resistance. In addition, high surface area, excellent electrical conductivity, and tunable surface chemistry make them suitable for tribological applications. These applications can be broadly categorized into solid lubricants, lubricant additives, and coatings. Before exploring the tribological application of MXene, it is also important to evaluate the friction and wear properties of the standalone MXene. The suitable tool to characterize the tribological property of nano-micro-sized MXene is atomic force microscopy (AFM). Zhou et al. [87] studied the friction and wear performance of two MXenes materials: Ti_3_C_2_ and Nb_2_C. The friction force is shown in Figure 10. Under a normal load of 4 μN, Ti_3_C_3_ yielded a frictional force of 800 nN that was reduced to ~600 nN, which is equivalent to 0.15 as the COF under 4 μN of normal force. For Nb_2_C, the frictional force under the same condition is ~200 nN, equivalent to ~0.05 COF. In addition, the authors also evaluated the effect of temperature on the frictional force and compared it with the performance of Mica. As the temperature increased from 25 to 40 °C, the decrease in frictional force was 61% and 92% for Ti_3_C_2_ and Nb_2_C, respectively. The authors also suggested that the lower surface dipole moment density of Nb_2_C yields a lower frictional force in comparison with Ti_3_C_2_. It is important to highlight that the observed COF from this AFM study is in the range of 0.15 to 0.05, which is similar to or lower than that of graphene, ~0.22 [88]. Although the tests were conducted under different conditions, the lower friction suggests that the MXene has suitable tribological properties that can be used in bulk material as a composite, a coating, or suspended in liquid lubricants.

Raman spectroscopy is a valuable tool in assessing MXenes’ structural integrity, especially under mechanical loading during tribological testing. Several studies have employed Raman spectroscopy to monitor oxidation progression, surface chemistry changes, and defect formation in MXene layers during wear and friction processes [89,90]. Sarycheva and Gogotsi have shown that the Raman spectra of Ti_3_C_2_T_x_ can provide synthesis-dependent variations, and the photoluminescent features can indicate degradation through the formation of TiO_2_ and amorphous carbon [91].

### 4.1. Solid Lubricants

MXenes have shown potential as solid lubricants for metal and polymer matrix composites due to their layered structure, which allows for easy shear and low friction between layers. This property is particularly advantageous in environments where liquid lubricants are impractical, such as in high-temperature or vacuum conditions. Studies have demonstrated that MXenes can significantly reduce friction and wear in various mechanical systems, making them effective solid lubricants. Recent research has focused on exploring the tribological performance of various MXene compositions and structures. For instance, studies have investigated the effects of different transition metals, surface functionalization, and synthesis methods on the tribological properties of MXenes.

**Figure 11:**
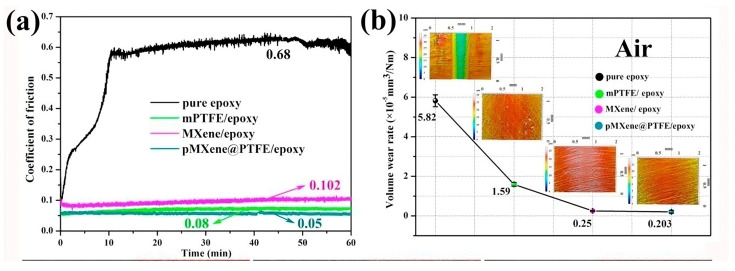
(**a**) COF and (**b**) wear volume of the epoxy, PTFE/epoxy, MXene/epoxy, and MXene@PTFE/epoxy [94].

The addition of MXene to the polymer matrix has also been advantageous for tribological properties. Zhang et al. [92] added the Ti_3_C_2_ to the UHMWPE matrix, and they observed that 2 wt.% Ti_3_C_2_ yielded the lowest COF ~0.13 which is ~31% lesser than UHMWPE with no MXene. The lower COF and wear results were due to the formation of a tribofilm consisting of MXene. Similarly, Gao et al. [93] added Ti_3_C_2_T_x_ into the epoxy matrix and formed a composite by curing the mixture. The authors also added nano-sized Al_2_O_3_ particles, and they observed that the mixture reinforcement of Al_2_O_3_ with Ti_3_C_2_T_x_ yielded an extremely low COF ~0.02 against steel in the presence of ultra-low sulfur diesel. The COF value is significantly lower than the COF values of epoxy (0.2) and epoxy + Ti_3_C_2_T_x_ (0.15). This extremely low COF is due to the formation of a composite tribofilm that consists of Al_2_O_3_, Ti_3_C_2_T_x,_ and amorphous carbon. However, the complex nature of the tribofilm makes it difficult to identify the contribution of MXene towards superior tribological properties. In another study on epoxy/MXene composites, positively charged Ti_3_C_2_T_x_ was coated on PTFE before synthesizing the epoxy composite [94]. The resulting COF and wear volume of the composites are shown in Figure 11a and Figure 11b, respectively. The combination of MXene with PTFE outperformed the individual additives in the epoxy matrix. The authors showed that the PTFE enables oxidation resistance in MXene and improves tribological performance, especially at high humid conditions (80% RH). Other studies on epoxy matrix composites are summarized in Table 5, which suggests that MXene is a suitable reinforcement material to improve tribological properties.

Other studies have also demonstrated the effectiveness of MXene as a solid lubricant. Liang et al. [99] showed that adding a low fraction of Ti_3_C_2_T_x_ with Polyimide/polyurea copolymers resulted in COF reductions of ~59% compared to pure polymer composites. In another study, PEEK/PTFE composites reinforced with Ti_3_C_2_T_x_ MXene achieved ultra-low COF (~0.065 ± 0.003) and a ~40% reduction in wear rate. This was facilitated by the formation of a transfer tribofilm of MXene, which selectively adheres PTFE to ZrO2 counter material [100].

### 4.2. Lubricant Additives

When used as additives in lubricants, MXenes can enhance the performance of conventional lubricants by reducing friction and wear. Their high surface area and functionalized surfaces enable strong interactions with lubricant molecules, improving the stability and efficacy of the lubricant. MXenes can also form protective tribofilms on sliding surfaces, further reducing wear and extending the lifespan of mechanical components.

Recent research has explored MXene dispersions in ionic liquids and bio-based lubricants. Incorporating 0.01 wt.% of Ti_3_C_2_T_x_ MXene with optimized interlayer spacing into outboard engine oil resulted in a ~14.5% reduction in the coefficient of friction and a ~6.3% decrease in wear scar diameter compared to the base oil [101]. Similarly, Neem seed oil–PAO6 blends with 0.08 wt% Ti_3_C_2_T_x_ demonstrated around a 10% COF reduction, with tribological performance comparable to that of conventional mineral oils [95]. Table 6 presents key findings where MXene is used as a lubricant additive for tribological applications.

### 4.3. Coatings

The application of MXene-based coatings on mechanical components can provide an effective barrier against wear and corrosion. Due to their unique two-dimensional structure and tunable surface chemistry, MXenes can be engineered to form dense adherent coatings with excellent mechanical properties. These coatings serve as effective barriers against wear, corrosion, and oxidation, thereby significantly extending the service life of mechanical parts. MXene coatings can be tailored to achieve high hardness, superior adhesion to various substrates, and remarkable wear resistance under dry or lubricated conditions. Moreover, their intrinsic high electrical and thermal conductivity enables the development of multifunctional surfaces that can dissipate heat efficiently or conduct electricity where needed. These properties make MXene coatings ideal for a broad range of industrial applications, including aerospace, automotive, electronics, and energy systems. Marian et al. [108] explored the effectiveness of Ti_3_AlC_2_ as a coating for 100Cr6 steel using the drop-casting deposition method. They have achieved a COF of ~0.26 at a Hertzian contact pressure of 1.47 GPa and 20% relative humidity. COF and wear volume were 2.3 and 2.7 times less than the uncoated surface. In another study, Zeng et al. [109] deposited Ti_3_C_2_T_x_ coatings on impregnated zinc phosphate graphite using electrophoretic deposition at 5 V, 10 V, and 15 V. The coating at 5 V showed the lowest COF, 0.18 at 200 °C, 0.25 at 300 °C, and 0.21 at 400 °C. This demonstrates the excellent high-temperature tribological performance of MXene-based coatings.

### 4.4. Emerging Applications in Triboelectronics

MXenes are increasingly recognized for their potential in triboelectronic systems, particularly as active components in triboelectric nanogenerators (TENGs) and self-powered sensors [110,111]. TENGs offer a promising route to self-powered devices by converting mechanical energy into electricity [112]. The unique combination of the high electrical conductivity, mechanical flexibility, and surface tunability of MXenes enables efficient charge transfer and robust contact electrification. Recent studies have demonstrated that functionalized Ti_3_C_2_T_x_ MXene films can significantly enhance the output performance of TENGs when used as either triboelectric layers or conductive electrodes [113]. The ability to integrate MXenes with polymers, elastomers, or textile substrates also offers opportunities for developing flexible and wearable triboelectric devices [114]. The ability of MXene to conform to curved surfaces and resist oxidation broadens its application in human–machine interfaces, soft robotics, and motion sensors [115].

## 5. Challenges, Opportunities, and Future Directions

### 5.1. Obstacles in Manufacturing and Usage

Despite their unique behavior and properties, there are still challenges to be overcome in the synthesis and application of MXenes. For instance, controlling the etching process to produce MXenes with desired properties is still a complex task that requires further research [116]. Additionally, scaling up the production of MXenes for commercial applications is another area that needs to be addressed. Another key concern is their environmental stability, which is quite low, like that of most 2D materials. MXenes tend to degrade in the presence of water and oxygen, which can limit their practical applications and effective use over time [117]. To make MXenes a viable composite for future uses, improvements have to be made in terms of their environmental stability. In doing so, MXenes conductivity and electronic structure, which are highly desired, can be maintained for their applications. This is a significant challenge that researchers in the field are actively working to address. The sooner this weakness can be addressed, the longer lasting these materials will be in their applications. Additionally, the synthesis of MXenes often involves the use of hazardous chemicals such as HF acids, which raises safety and environmental concerns. Workers will be subjected to these elements, resulting in more concerns being brought up in industrial applications. These issues need to be addressed in the production phase of MXenes.

The scalability (and sustainability) of MXene production is another significant challenge. While the laboratory-scale synthesis of MXenes has seen substantial progress, scaling these processes for industrial applications remains a challenging task. The complexity and high cost of current synthesis methods further exacerbate this issue, leaving most of the manufacturing industry hesitant to make these revolutionary changes. To mitigate these concerns, research is being directed towards developing simpler, cost-effective, and scalable synthesis methods [118,119]. Additional safety concerns are another contributing factor, as the use and disposal of chemical waste must be accounted for. For more manufacturers to switch to using MXene technology, there needs to be further development of safer and more environmentally friendly synthesis methods.

MXenes have always been an asset to new and innovative inventions such as electrical storage and biomedical devices. Although MXenes are a dream come true, they have their inherent flaws. This includes but is not limited to the lack of essential materials, delamination processes, surface contaminations, manufacturing scalability, post-synthesis processing, low stability, and more. One example of the lack of precursor materials is that there is a highly competitive field for raw materials to synthesize MXene, which results in a reduction in research [120]. Not only does this make it difficult to manufacture a larger quantity of quality MXenes, but it also reduces the knowledge of the products. The low stability in these nanomaterials is also another obstacle that inhibits the smooth manufacturing of MXenes, as the moisture of air or water limits the long-term storage of their intended uses [121]. This problem may cause system-wide failures in electrical, mechanical, and biomedical appliances, which is a hazard to the public. This brings up the subject of surface contamination in the carbides as unwanted particles, oxidation, and radioactivity may occur sub-automatically. Achieving stable MXene status is no easy task, as more properties come with more complex MXenes, which would require more research. In the event of theoretical stability amongst these 2D carbides, one would have to engineer and manufacture a wide scale of MXenes, which can be difficult [122].

MXenes may not just have obstacles during manufacturing, but also during their intended uses, which creates an abundance of problems. Technologies such as energy storage, supercapacitors, medical devices, and sensors all have MXenes that could potentially malfunction during operation. According to an article by Chen et al. [123], MXenes face challenges related to their interaction with intercalated species, as the intercalation chemistry and physics can significantly influence their fundamental structural and functional properties. These interactions may lead to alterations in layer spacing, electronic configuration, and surface chemistry, thereby impacting the overall performance and stability of MXene-based materials in various applications. Similarly, Ti_3_C_2_T_x_ MXene is being extensively explored for use in piezoelectric applications. However, during functionalization with surface groups, the material often experiences the loss of its atomic symmetry [124]. Even though these piezoelectric devices have trouble maintaining Ti_3_C_2_T_x_ atomic stability, innovative technologies are always growing and will pave new methods for improving piezoelectric devices. Another industry that is experiencing difficulties utilizing MXenes is the direct ink writing technologies; 2D/3D printing machines are developed to produce super capacitors in the energy industry. However, these machines need a large amount of MXenes to function properly, as failing to do so will result in printed layers becoming self-restacking during their drying phase [125]. To mitigate this, researchers often incorporate additives like cellulose nanofibers or conductive spacers to maintain dispersion, prevent self-restacking, and preserve electrochemical functionality in the printed layers [125,126]. Despite the numerous difficulties in manufacturing MXenes and their practical use, MXenes have many positive effects that outweigh their flaws. Once these hurdles are overcome, MXenes hold the potential to revolutionize manufacturing with their unique combination of properties, making them a compelling option for a wide range of industries.

### 5.2. Safety and Environmental Factors

MXenes are relatively new materials being introduced into manufacturing. MXenes are generally considered to be safe materials, as they are chemically stable in various environments [127]. However, some safety and environmental factors need to be considered when working with them, for instance, checking for their toxicity. Although many MXenes exhibit good biocompatibility, the toxicity of specific MXene compounds can vary [128]. While some MXenes have demonstrated biocompatibility in certain applications, their potential toxicity is an area of active research. Biocompatibility studies are crucial to assess the effects of MXenes on living organisms [129]. Factors such as the specific composition, surface functionalization, and size of MXene particles can influence their toxicity [130]. Understanding the interactions between MXenes and biological systems can help researchers identify any potential health risks and develop appropriate safety guidelines for their use in various applications. That is why it is important to conduct thorough toxicity studies to assess the potential risks associated with their usage.

Another safety factor that needs to be taken into consideration is exposure during synthesis. For example, strong acids, such as HF acid, are commonly used in the etching process to produce MXenes, and they can pose significant health risks if mishandled [131]. High-temperature processes involved in the synthesis may also generate harmful byproducts. That is why strict safety protocols, such as fume hoods, gloves, and protective clothing, are essential to minimize the risk of exposure to hazardous substances.

Environmental impacts for MXene or other nanomaterials have the potential to affect ecosystems if they are released into the environment. Understanding their behavior in various environmental matrices, such as water and soil, is crucial to assessing their potential impact on aquatic life and soil organisms [132]. Studies on the long-term stability, bioaccumulation potential, and degradation pathways of MXenes in different environmental conditions can help researchers evaluate their environmental safety and develop strategies to minimize any adverse effects [133]. That is why continued research focused on understanding the toxicity, synthesizing safely, and the environmental impact of MXenes is essential for ensuring their responsible and sustainable use in various applications, while minimizing potential risks to human health and the environment.

### 5.3. Opportunities and Future Directions

Despite the challenges, MXenes present many opportunities for future materials science and manufacturing technologies. Their high conductivity and large surface areas, doubled with their layer nanostructure, grant MXenes desirable traits for various electronics, aerospace, and mechanical applications. MXenes are highly conductive, making them excellent materials for energy storage applications. They also demonstrate significant potential in supercapacitors and batteries, offering high capacity and excellent rate performance [134]. As improvements are made in maintaining optimal structure qualities in MXenes, these traits can be harnessed to their full potential, creating endless fields in which the material can enhance existing technologies.

Tunable surface chemistry is another exciting aspect of MXenes. By modifying the surface functional groups, the properties of MXenes can be tailored to suit specific applications. This versatility opens a wide range of possibilities in various fields, including catalysis, sensing, and environmental remediation. Research is already being completed to see whether MXenes can be applied to filters to remove pollutants and unsafe elements from saline water, potentially allowing for purified ocean water [135]. This could be a game changer in addressing the global water crisis, providing a sustainable solution for clean water production. The tunability of MXenes is a powerful feature that offers many opportunities across various fields. As research is continued and we further understand these unique characteristics of MXenes, we can look forward to innovating solutions that fully harness their potential. The exploration of MXenes in these realms is a testament to their versatility and the vast potential they hold for future technological advancements.

Looking at the future of MXenes, there are several promising directions for continued research. Developing environmentally friendly and scalable synthesis methods could address current challenges associated with MXene production. Such approaches would not only improve the environmental sustainability of MXenes, but also make their large-scale manufacturing more feasible. Another important area is the exploration of new compositions and structures, as the size and nature of compositional combinations can vary significantly. With a wide range of transition metals and carbonitride configurations available, the potential to discover new MXenes with unique and useful properties remains largely untapped.

## 6. Conclusions

MXenes are an exciting new class of 2D materials that are quickly gaining attention for their wide-ranging potential in science and engineering. Synthesized by selectively etching and delaminating layered MAX phase precursors, MXenes naturally develop surface groups like -F, -OH, and -O during synthesis. These give them strong hydrophilic properties, allowing them to form stable surfactant-free dispersions in water. As a result, it is relatively easy to create MXene-based films and paper-like electrodes.

Recent developments in synthesis strategies, including minimally toxic and fluoride-free etching methods, have opened pathways for safer scalable production, while also offering better control over surface terminations and interlayer chemistry. These trends are crucial in tailoring MXene properties for specific applications.

What truly sets MXenes apart is their remarkable combination of thermal, electrical, and optical properties, which can be tailored through surface chemistry and interlayer modifications. For example, Ti_3_C_2_T_x_ exhibits metallic conductivity when intercalated with water or surface terminations, while other MXenes like Mo_2_CT_x_ display semiconducting behavior, demonstrating the tunability of their electronic properties. In composite and film form, MXenes also offer impressive mechanical strength and flexibility, making them suitable for use in flexible electronics and wearable devices.

This review further emphasizes MXenes’ growing role in tribological and triboelectronic applications, where their structural integrity, electrical performance, and interfacial behavior under stress have shown promising results. While many of their theoretical properties have been predicted through simulations, continued experimental research is expanding the understanding of MXenes’ full capabilities. With their versatile and tunable properties, MXenes are emerging as key materials in next-generation technologies.

## Figures and Tables

**Table 1:** The search criteria used for the systematic literature analysis with the Web of Science [12].

Database Used for the Search	Web of Science
Keywords Used for the Search	(1) MXene
	(2) MXenes
	(3) MAX Phase Etching
	(4) MXene Synthesis
	(5) MXene Fabrication
	(6) MXene Properties
Timespan of Search	January 2011–December 2024
Language	Any
Types of Research	Review and Research Journal Publications, Books, Book Chapters, and Patents

**Table 2:** A summarization of the different techniques used for MXene fabrication/manufacturing.

MXene Manufacturing Route	Summarization	Parameters That Control Structural Features	References
HF Etching	This process consists of selectively dissolving the A element of the MAX phase through an HF solution	Parameters include etching time, the concentration of HF, and the operational temperature	[25,27,28,29,30,31,32,33,34,35,36,37]
Molten Salt Etching	This process consists of utilizing molten salts to chemically react with the A element of the MAX phase	Parameters include the chemistry of the molten salts, the temperature of the molten salts, and the submersion time	[38,39,40,41,42,43,44,45]
Electrochemical Etching	This process consists of inducing a chemical reaction with the MAX phase and selectively removing the A element	Parameters include the electrolyte solution, etching time, current density, and voltage	[37,40,46,47]
Chemical Vapor Deposition	This process consists of using CVD to allow for metal precursors to react with gases to form the MXenes	Parameters include operational temperature, the gas flow rate, the concentration of the precursors, and the operational pressure	[38,48,49,50,51]

**Table 3:** Comparative analysis of MXene synthesis methods [34,37,38,40,46,47,51,60].

Synthesis Method	Yield	Scalability	Environmental Impact	Cost	Advantages	Limitations
HF Etching	High	Moderate	High (toxic HF handling)	Low to moderate	Widely used, high-quality MXenes	Hazardous chemicals, disposal issues
Molten Salt Etching	Moderate	Moderate	Moderate	Moderate	Fluoride-free; accordion-like morphology	Requires high temperature; salt recovery needed
Electrochemical Etching	Moderate	High	Low	Low	Environmentally friendly; tunable structure	Longer processing time; uniformity issues
Chemical Vapor Deposition	Variable	Low to moderate	Low	High	Fluoride-free; direct synthesis of 2D sheets	Expensive setup; not yet widely adopted
Fluoride and Acid-Free Etching	Moderate	High	Very low	Low	Green, scalable, safe	Under development, limited types

**Table 4:** Chemical reactivity by termination type.

Termination	Reactivity Characteristics	Best Applications
-O	Lewis acidic, stable	Oxidation reactions
-OH	Bronsted basic, hydrophilic	Biomolecule adsorption
-F	Electron-withdrawing, hydrophobic	Fluorination reactions
Mixed	Tunable acid-based properties	Multifunctional catalysis

**Table 5:** Summary of tribological studies on epoxy matrix composites.

MXene Material	Matrix	Synthesis Method	Tribological Testing	Ref.
Ti_3_C_2_T_x_ (3 wt.%)	Aluminum	Pressureless sintering followed by extrusion	COF ~0.2 at 5 N normal load against GCr15 steel ball (~2.5 times reduction)	[95]
Ti_3_C_2_T_x_ (15%)	Ni_3_Al	Sparks plasma sintering	COF = ~0.5 (29% reduction) and wear rate = 1.64 × 10^−4^ mm^3^/m (93% reduction)	[96]
Ti_3_C_2_ (2 wt.%)	UHMWPE	Pressure molded	COF~0.13 (31% reduction)	[92]
Ti_3_C_2_T_x_ (3 wt.%) by HF leaching method	Epoxy + 3% Al_2_O_3_	Curing	COF ~0.02 against steel (COF of epoxy + 3% Al_2_O_3_ = 0.07	[93]
Ti_3_C_2_T_x_ (0.5 wt.%)	Epoxy	Curing	COF ~0.65 (28% reduction) and wear rate reduced by 79%	[97]
Ti_3_C_2_T_x_ (1 wt.%) by HF leaching with ultrasound-assisted delamination	Epoxy	Curing	COF = 0.65 (26% less) and wear 6.61 × 10^−14^ m^3^/N·m (63% lesser)	[98]
Ti_3_C_2_T_x_ coated on PTFE particles	Epoxy	Curing		[94]

**Table 6:** MXene as a lubricant additive for tribological applications.

MXene	Base Lubricant	Result	Ref.
Ti_3_C_2_T_x_ + N, N-dimethylformamide	5750 oil	Reduction in wear rate by 92% under 100 N against GCr15 bearing steel	[101]
Ti_3_C_2_ (2 wt.%)	Lithium grease	COF = 0.045 and wear rate = 5.8 × 10^−8^ mm^3^N^−1^m^−1^ reduced by 56.7% and 26.6% compared to MoS2 lithium grease for 42CrMo steel and E52100 steel tribo pair	[102]
hexadecylphosphonic acid grafted Ti_3_C_2_T_x_ + zinc dialkyl dithiophosphate (ZDDP)	Polyalkylene glycol	COF ~0.1 (33% reduction)	[103]
tetradecylphosphonic acid (TDPA) modified Ti_3_C_2_T_X_ + Graphene oxide	Polyalphaolefins base oil	COF = 0.09 (54% reduction) and wear rate = 4.4 × 10^–6^ mm^3^/Nm (90.0% reduction) for steel to steel tribo pair	[104]
Ti_3_C_2_T_x_ with in situ grown SiO_2_	Mineral oil (500 SN)	COF = 0.1 (44% reduction), and wear reduced by 73%	[105]
Ti_3_C_2_T_x_ grafted on ionic liquid (1-aminoethyl-3-methylimidazolium bromide [NH_2_C_2_MIm] [Br])	Mineral oil (500 SN)	COF = 0.12 (40% reduction) and 81% reduction in wear volume for AISI 52100 steel tribo pair	[106]
Ti_3_C_2_T_x_ MXenes modified with dodecyl phosphonic acid	OSP-46	COF = 0.09 (24% reduction) and 85% reduction in wear rate for AISI-52100 GCr15 steel tribo pair	[107]

## Data Availability

No new data were created or analyzed in this study. Data sharing is not applicable to this article.
